# Grafts

**Published:** 1890-11

**Authors:** W. A. Yingling

**Affiliations:** Nonchalanta, Ness County, Kansas


### GRAFTS.

NONCHALANTA, NESS COUNTY, KANSAS, Sept. 30th, 1890.

EDITORS HOMŒOPATHIC PHYSICIAN:—What is your idea of the two articles in the September number of the *Homœopathic Recorder*, on "How Hahnemann cured," and "Grafts"? Such articles tend to produce confusion and deter the novitiate from entering the field of sure cure. I have but little experience with grafts, and was very slow even in trying them, but I thought no one could convince me either way as well as experience, so I sent after twenty-two remedies. The result has been satisfactory so far as tried. One case where I used the graft of Cina$^{10m}$ I consider remarkable. A child was extremely low, picking nose, etc. Parents had *never seen any worms pass per anus.* I gave the child Cina$^{3}$, no results. In fourteen days I gave Cina$^{10m}$ from graft, and was surprised to find many worms, pieces of all sizes and shapes, pass from the child within a day or two. In about ten days all indications of worms per rectum were absent. One more dose of the same graft, Cina, brought more worms from the child, and with its action most decided improvement in every way. I gave Onosmodium$^{3}$, and same, 30, during a long time for deafness with dryness of ears; no secretion whatever; especially deafness for the human voice; could hear the clock tick during laughter and conversation, but very difficult to hear a voice, unless very distinct. The 3 and 30 gave no benefit. I sent after Onosmodium$^{cm}$ graft, and, behold! the ear begins to secrete, the noises cease, hearing *much* improved, as well as the general health. This is the result of two doses ten days apart. I shall continue the remedy with the expectation of final recovery. I am using from the 200 to the 1,000 principally, but the results of the grafts in the CM have been so brilliant I shall continue my investigation. In such matters I take no man's word, but investigate for myself. To make sure of the result I am careful to get the exact remedy instead of guessing, and then opposing either high potencies or grafts. My 200 to 1,000 I get from Bœricke & Tafel in fluid form. My few grafts I get from Dr. Swan.

Until a year past I used nothing higher than the 12x. Now I am so well pleased and find such wonderful results from the high potencies that I use nothing less than the 200. I had a case of threatened abortion and made a test of the high potencies. The case, second pregnancy, third month, had severe pains, a great loss of blood, and *could not move without the flow increasing; pains from the back to the pubis*, etc., Sabina$^{1000}$ (B. & T.) one dose in water entirely cured, and the woman is now doing her own work, and has no bad result whatever from the trouble.

Before I had the high potencies I dreaded labor cases. Now I have no trouble with them. My mountain fever cases get along without a crisis, whereas I could not do this with the low potencies. With such an experience, though very short, who could or would go back to the flesh-pots of Egypt in the form of allopathic palliatives, or to the slow acting and uncertain low potencies? The reason so many young men have the high potencies and remain in the valleys of the low, is the want of sufficient knowledge to use the high. It requires a much *closer* prescribing to derive any benefit from the high. If I did not know what to give, I should never give a high potency. It does not make so much difference with the low, yet Sac-lac. is the best remedy in an uncertainty. Whilst I am still investigating the graft question without a definitive opinion as yet, I have no doubt as to the high potencies being the *very best* for promptness, effectiveness, and enduring action.

I think you should speak on the question of grafts and Hahnemannian high potencies without reserve. Many young men are on the fence undecided, and will watch for the articles for the journals professing to teach pure Homœopathy. Sharpen your quill, dip it in the fluid of truth and experience and write. Blow your horn of eternal truth without fear or favor. What we want is *the* truth, whatever that may be.

Yours truly,

W. A. Yingling.

